# Aortic propagation velocity in predicting coronary artery disease: A systematic review and meta-analysis

**DOI:** 10.1097/MD.0000000000034243

**Published:** 2023-07-21

**Authors:** Fereshteh Ghaderi, Shabnam Niroomand, Hoorak Poorzand, Hedieh Alimi, Laila Bigdelu, Afsoon Fazlinezhad, Faeze Keihanian

**Affiliations:** a Cardiovascular Department, Echocardiography Laboratory, Faculty of Medicine, Mashhad University of Medical Science, Mashhad, Iran; b Community Medicine Department, Faculty of Medicine, Mashhad University of Medical Science, Mashhad, Iran.

**Keywords:** aortic propagation velocity, arterial stiffness, coronary artery disease, echocardiography

## Abstract

Coronary artery disease (CAD) and its outcome, myocardial infarction, is yet a significant etiology of mortality and morbidity nowadays. The aortic propagation velocity (APV) can be a simple, straightforward and novel echocardiographic index for the risk stratification in the evaluation of CAD. In this meta-analysis, we evaluated the predictive role of APV in CAD.

**Material and methods:**

Relevant electronic bibliographies (PubMed, ScienceDirect, Scopus, EMbase, the Cochrane library) were explored. Related reports were selected according to the inclusion and exclusion criteria. Meta-analysis was performed using the Comprehensive Meta-analysis 2.0 software.

**Results:**

Eventually, 5 articles met the inclusion criteria and included in the meta-analysis. Five studies with 490 patients reported the APV mean in CAD and non-CAD groups. A random-effect model was used and the pooled findings demonstrated a significant higher APV in non-CAD group compared to CAD group (SMD: 2.39, 95% CI: 1.70–3.07, *P* < .001, *I*^2^: 84%, Q: 19.03). The diagnostic value of APV in predicting CAD showed 86.3% sensitivity (95% CI: 74–91, *P* value < .001, *I*^2^: 65%, Q: 8.53, *P* value: .03) and 83.8% specificity (95% CI: 69–94, *P* value < .001, *I*^2^: 60%, Q: 9.89, *P* value: .01).

**Conclusion:**

There was a predictive role of APV in CAD with suitable specificity and sensitivity. Moreover, aortic distensibility and aortic strain were significantly different in CAD and non-CAD patients. APV could be used as a good noninvasive tool for screening CAD.

## 1. Introduction

Arterial wall stiffness is an index of vascular aging and an important cause of predisposing patients to atherosclerosis and correlated with cardiovascular events.^[1]^ Coronary artery disease (CAD) and its outcome i.e. myocardial infarction, is yet a significant etiology of mortality and morbidity nowadays. Optimal risk stratification of patients with acute myocardial infarction has a critical role in suitable care of patients. Risk prediction can be performed according to clinical, electrocardiography, two-dimensional echocardiography and biochemical parameters.

Atherosclerosis is a complex, chronic and multi-factorial disease, which can affect the whole arterial system.^[2]^ Atherosclerotic plaque starts early in life and grows slowly over decades. Although atherosclerosis is almost common in the modern world, most plaque remains asymptomatic lifelong. Despite systemic tendency of some patients to plaque erosion independent of conventional risk factors, traditional risk factors and consequent chronic inflammation lead to the development of atherosclerosis.^[3]^ Collagen and elastin are the 2 major effective proteins in the vascular wall establishment and flexibility. Their content is balanced in a dynamic process of production/degradation. If this balance is altered by any trigger of inflammatory process, it leads to an increase of abnormal collagen and a decrease in normal elastin quantity. Several cardiovascular risk factor such as aging, hypertension, diabetes mellitus, and chronic kidney disease contributed in over-production of collagen, considering their different times of exposure.^[4]^ Another mechanism for stiffening of arterial wall is endothelial dysfunction, caused by changes in endothelial cell signals and cell tone of smooth muscles of vessels, further to structural alterations.^[5]^ Endothelial dysfunction is secondary to various factors such as any local imbalance between vasodilator nitric oxide and vasoconstrictors, increased expression of asymmetrical dimethylarginine, and activation of reactive oxygen species.

Variables like aortic strain (AS), aortic distensibility (AD), pulse pressure, and augmentation index, and pulse wave propagation velocity were recently applied to determine aortic stiffness.^[6–8]^ By aging, CAD risk factors accumulate and vessel flexibility decreases. The aortic propagation velocity (APV) might be decreased with growing arterial stiffness and lowering strain and distensibility.^[9]^

APV can be a simple, easy and novel echocardiographic parameter for the risk stratification in the evaluation of CAD. The purpose of this meta-analysis was to assess the predictive value of APV in CAD as an index of arterial stiffness.

## 2. Methods

### 2.1. Literature search

Relevant electronic databases (PubMed, ScienceDirect, Scopus, EMbase, the Cochrane library) were searched (up to November 2022) using the following terms: CAD, APV, echocardiography, aortic stiffness, atherosclerosis, and aortic velocity). Literature selecting was performed by reading the article title and abstract, eliminated the studies not meeting the inclusion/exclusion criteria. For those that were more ambiguous, full text was accessed and then a choice was made. General data includes published information, patient information, the quality information, the index and data of result etc. was extracted. Literature selecting and data extraction processes were independent, then the results were cross-checked.

### 2.2. Object and standard

#### 2.2.1. Inclusion and exclusion criteria.

The inclusion criterion was cross sectional studies that used APV index in predicting CAD. Studies were excluded if APV was assessed for another purpose like other specific types of angina pectoralis or congenital heart disease, and repeated literatures. Only English literatures were used.

#### 2.2.2. Object of study.

CAD diagnosis was based on medical history of documented myocardial infarction, coronary revascularization or epicardial CAD detected during coronary angiography for symptomatic patients or typical electrocardiographic changes.

### 2.3. Data extraction

Two reviewers independently extracted data and reached a consensus on all items. The following information was achieved from each study: the first author’s name, publication date, ethnicity, population, characteristic of target population, treatment protocol, definition of cases, outcomes and study period.

### 2.4. Study outcomes

Aortic propagation velocity (APV), left ventricular ejection fraction, AS, and aortic dispensability (AD) were studied. All parameters were defined in a previously published study.^[9]^

### 2.5. Assessment of methodological quality

Quality control was performed by 2 independent authors based on the Strengthening the Reporting of Observational Studies in Epidemiology checklist for cross sectional studies.^[10,11]^ Any disagreement was checked by a third reviewer. We used these items for study assessment: the study ability to correctly respond to study question; well-defined sampling method (for both the subjects and setting); enough sample size; checking for selection bias; accurately outcome measurement; accurately outcome analysis; reporting the findings with confidence interval (CI); and rationalizing the conclusion according to the findings. In accordance with the appraiser agreement, a response of “yes” demonstrated low risk of bias and coded as “1” and “no” indicated high risk of bias and coded as “0.” If an item was unclear, it means an unknown risk of bias. A score of 0 to 8 was assigned to each investigation. Studies that achieved 6 or more scores were rated as high quality, while 4 to 5 was considered as moderate quality.

### 2.6. Statistical analysis

Analyzed the clinical and methodological heterogeneity of the included studies, used χ^2^ test and *I*^2^ test to judge statistical heterogeneity. When *P* > .1, *I*^2^ < 50%, and each study did not show significant heterogeneity, we used the fixed effect model. When *P* ≤ .1, *I*^2^ ≥ 50%, and each study showed significant heterogeneity, we made the subgroup analysis (according to the possible factors of heterogeneity) or sensitivity analysis. If the heterogeneity still existed and data based on the clinical significance view could be merged, we used the random effect model and explain the results cautiously. Continuous variables used the standard mean difference (SMD) as analysis statistics. 95% confidence interval (95% CI) was be used as effective size for the combined analysis. Hypothesis testing was carried out with u test, which was represented by *Z* and *P*. When *P* ≤ .05, it indicated that there was a significant difference between the 2 groups. Heterogeneity of the results between studies was assessed graphically by forest plots and statistically using the quantity *I*^2^ that describes the percentage of total variation across studies attributable to heterogeneity rather than chance. Statistical analyses were performed using Comprehensive Meta-analysis software version 2 (Biostat Inc., Englewood, NJ).

## 3. Results

### 3.1. Search results

**Figure 1.**
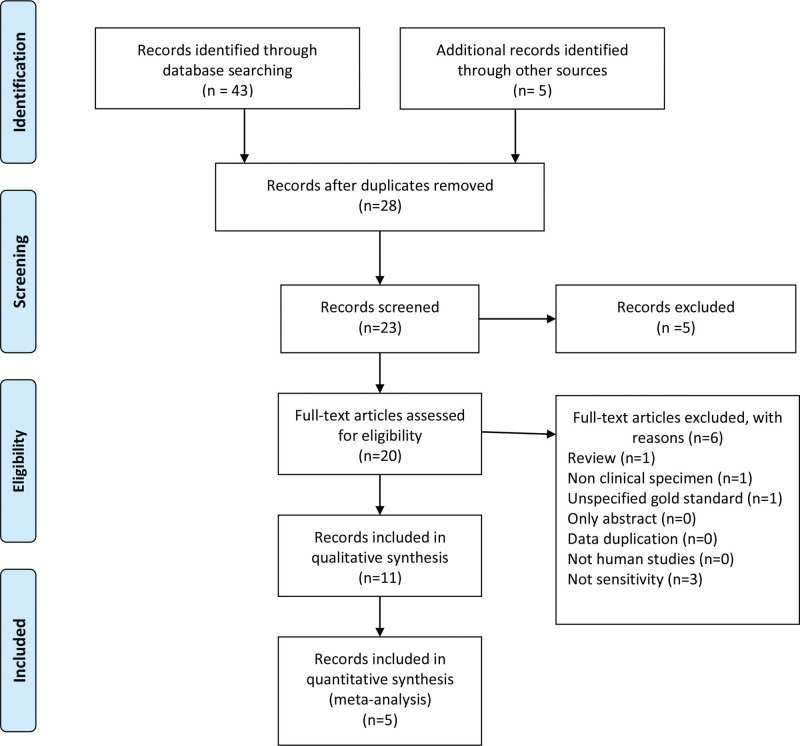
PRISMA flow diagram of literature selection.

A total of 20 applicable studies were recognized according to primary search criteria. Eleven irrelevant studies were excluded by initial screening of titles and abstracts. Gray studies were not found and no additional articles were added after the reference review. Finally, 5 articles met the inclusion criteria and entered the meta-analysis. The search process and literature selection are demonstrated in a PRISMA flow diagram (Fig. 1).

### 3.2. Study characteristics

In total, these studies included 299 participants in CAD groups and 191 in non-CAD groups. The sample size of these studies ranged from 70 to 127. Eligible studies were published between 2008 and 2022 and performed in India, Turkey, and Iran. All 5 studies were designed as cross-sectional and echocardiographic parameters including APV, AS, and AD were compared between CAD and non-CAD groups. A definitive diagnosis of CAD was performed based on the results of coronary angiography. The demographics and characteristics of the included studies are demonstrated in Table 1.

**Table 1:** Baseline characteristics of included studies.

Study	Chetty et al^[12]^	Gunes et al^[13]^	Sen et al^[14]^	Ghaderi et al^[9]^	Vamsikrishna et al^[15]^
Year	2016	2008	2013	2018	2022
Location	India	Turkey	Turkey	Iran	India
CAD/male	69/57	91/73	51/25	38/23	50/37
Non-CAD/male	31/3	36/18	42/26	32/13	50/33
Mean age ± CI					
CAD	54.81 ± 9.4	58 ± 9.6	55.2 ± 8.9	58.47 ± 13.29	54.00 ± 10.8
Non-CAD	54.13 ± 10.3	53 ± 11.8	52.3 ± 9	58.12 ± 9.65	51.10 ± 7.37
APV					
CAD	41.65 ± 4.94	28.3 ± 10.4	39.2 ± 13.9	48.63 ± 10.31	54.30 ± 14.75
Non-CAD	49.72 ± 6.38	57.3 ± 9.1	81.4 ± 21.4	77.75 ± 9.97	67.30 ± 10.47
LVEF (%)					
CAD	50.16 ± 10.3	54.4 ± 7	59 ± 6.9	56.34 ± 2.79	48.50 ± 6.30
Non-CAD	54.97 ± 8.95	58.9 ± 6.6	62.3 ± 5.6	56.56 ± 2.36	60.50 ± 7.30
AS (%)					
CAD	–	8.8 ± 5	7.4 ± 3.3	6.23 ± 1.93	3.28 ± 1.12
Non-CAD	–	7.4 ± 3.2	12.4 ± 5.4	11.66 ± 4.86	4.08 ± 1.02
AD					
CAD	–	3.9 ± 2.2	2.46 ± 1.63	2.46 ± 0.91	1.54 ± 0.60
Non-CAD	–	3.3 ± 1.7	4.17 ± 2.39	5.57 ± 2.25	2.02 ± 0.68
APV sensitivity	76%	82.4%	90.5%	96.9%	72.5%
APV specificity	72%	97.2%	92.2%	78.9%	62%

AD *=* aortic dispensability, APV *=* aortic propagation velocity, AS *=* aortic strain, CAD *=* coronary artery disease, CI *=* confidence interval, LVEF *=* left ventricular ejection fraction.

### 3.3. Risk of bias assessment

The methodological quality of the included studies is shown in Table 2.

**Table 2.**
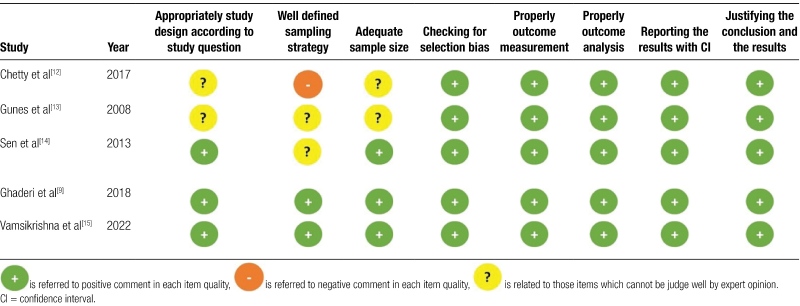
Methodological quality of investigations.

### 3.4. Outcomes for meta-analysis

#### 3.4.1. APV in CAD and non-CAD groups.

**Figure 2.**
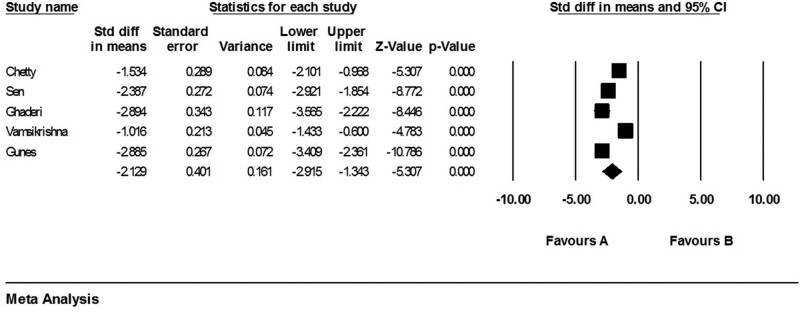
Forest plot representing standardized mean differences and confidence intervals (CIs) of APV in CAD and non-CAD groups. APV = aortic propagation velocity, CAD = coronary artery disease.

Five studies with 490 patients reported the APV mean in CAD and non-CAD groups. A random effect model was used and the pooled results demonstrated a significant higher APV in non-CAD group compared to CAD group (SMD: 1.97, 95% CI: 1.73–2.20, *P* value < .001, *I*^2^: 84%, Q: 19.03) (Fig. 2).

#### 3.4.2. The diagnostic value of APV.

**Figure 3.**
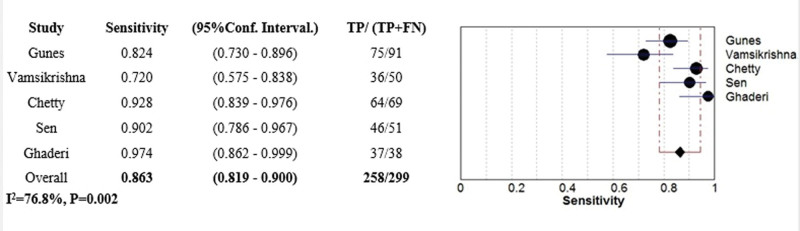
Forest plot representing sensitivity of APV in predicting CAD. APV = aortic propagation velocity, CAD = coronary artery disease.

**Figure 4.**
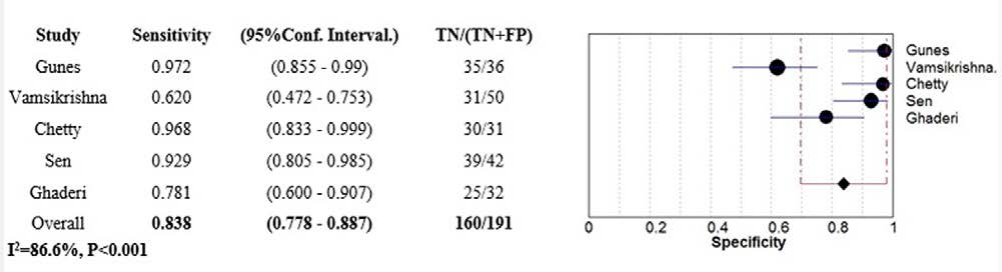
Forest plot representing specificity of APV in predicting CAD. APV = aortic propagation velocity, CAD = coronary artery disease.

**Figure 5.**
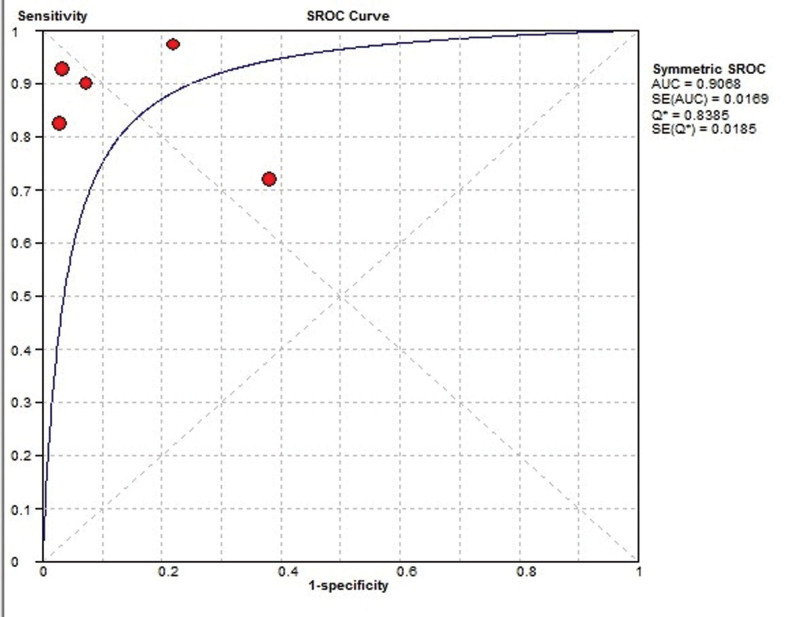
ROC curve of diagnostic value of APV in predicting CAD. APV = aortic propagation velocity, CAD = coronary artery disease.

The diagnostic value of APV in predicting CAD was assessed in all 5 articles with 490 patients. Combined results revealed that APV can estimate CAD with 86.3% sensitivity (95% CI: 0.81–0.90%, *I*^2^: 76.8%, Q: 8.53, *P* value: .002) and 83.8% specificity (95% CI: 0.77–0.88%, *I*^2^: 86.6%, Q: 9.89, *P* value < .001) (Figs. 3–5).

#### 3.4.3. AD in CAD and non-CAD groups.

**Figure 6.**
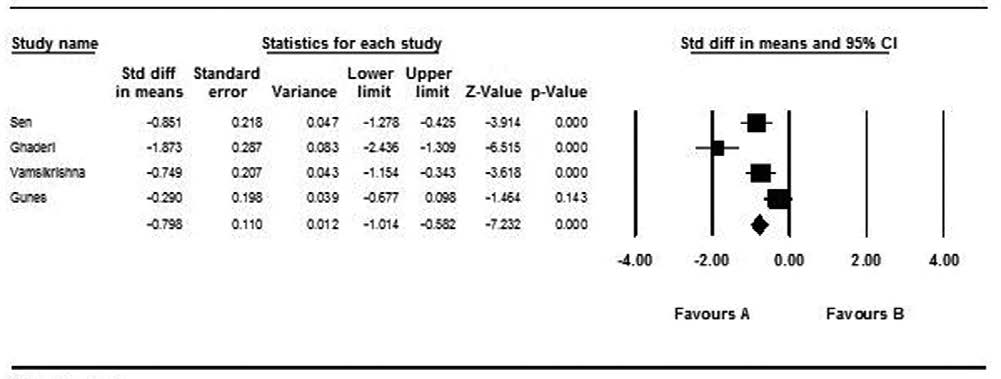
Forest plot representing standardized mean differences and confidence intervals (CIs) of AD in CAD and non-CAD groups. AD = aortic distensibility, CAD = coronary artery disease.

Four studies with 390 patients showed the mean of AD in CAD and non-CAD groups. A random effect model was used (*I*^2^: 95%, Q: 41.1, *P* value < .001). There was statistically significant difference in mean of AD between CAD and non-CAD groups (SMD: −0.798, CI: −1.024 to −0.582, *P* value: .001) (Fig. 6).

#### 3.4.4. AS in CAD and non-CAD groups.

**Figure 7.**
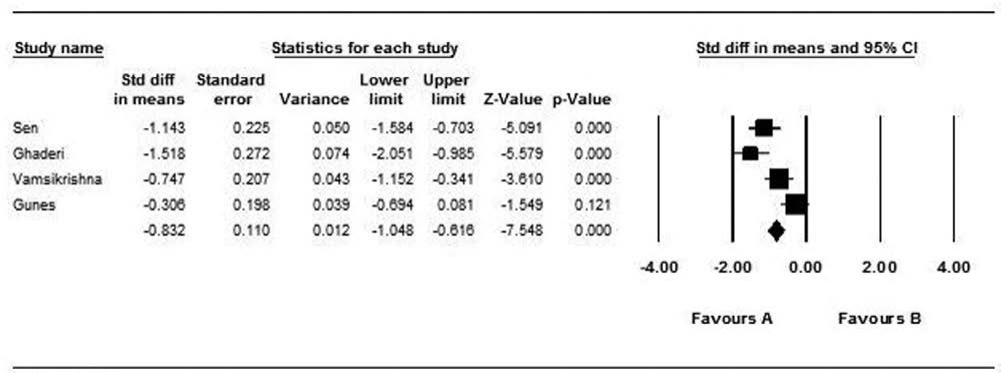
Forest plot representing standardized mean differences and confidence intervals (CIs) of AS in CAD and non-CAD groups. AS = aortic strain, CAD = coronary artery disease.

Four studies with 390 patients showed the mean of AS in CAD and non-CAD groups. A random effect model was used (*I*^2^: 95%, Q: 15.50, *P* value < .001). AS was significant lower in CAD group rather than non-CAD group, statistically (SMD: −0.832, CI: −1.048 to −0.616, *P* value: .001) (Fig. 7).

#### 3.4.5. Correlation of APV with SYNTAX score.

**Figure 8.**
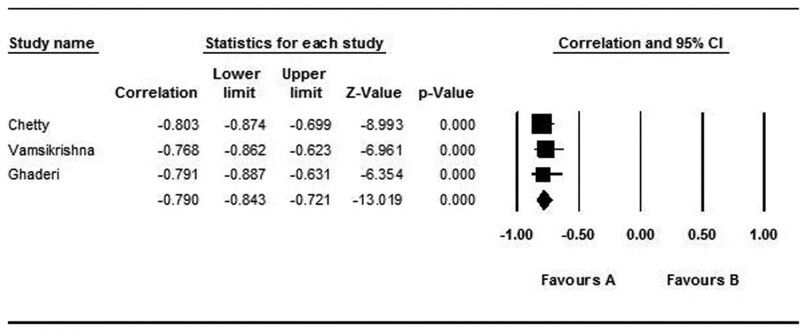
Forest plot representing correlation coefficient and confidence interval (CIs) of APV with SYNTAX score. APV = aortic propagation velocity.

Three studies with 157 patients revealed the correlation of APV with SYNTAX score. A random effect model was used (Q: 0.231, *P* value < .001). APV was adversely correlated with SYNTAX score (*r*: −0.790, 95% CI: −0.843 to −0.721, *P* value: .001) (Fig. 8).

## 4. Discussion

To best of our knowledge, this is the first investigation to evaluate the effects of APV in predicting CAD. Our results showed that lower APV could significantly distinguish CAD from non-CAD patients. It has an 86.3% sensitivity and 83.8% specificity in diagnosis of CAD. Moreover, AD and AS were significantly different between CAD and non-CAD groups. Another major finding was negative significant correlation between SYNTAX score and APV.

Arterial stiffness is a major predictor of cardiovascular events and all-cause mortality,^[16]^ even in subjects with no symptoms of overt CVD.^[17]^ Moreover, the quality of hypertension management and difference between various kinds of anti-hypertensive drugs can be directly assessed by measuring arterial stiffness.^[18]^ As atherosclerosis progresses, AD and AS diminish. Atherosclerosis decreases the arterial wall consistency and the aorta flexibility.^[16]^ By atherosclerosis progression, tunica media would be thicker and stiffer.^[19]^ Consequently, it is very important to determine atherosclerosis prior to be presented, non-invasively.

APV is one of the easiest echocardiographic parameters that correlates with significant CAD and aortic stiffness and distensibility. While aortic stiffness and AD are more correlated with conventional risk factors such as hypertension and increased age, APV correlates much with the extent of CAD and left ventricular dysfunction. As the severity of LV dysfunction increases, the APV decreases. It can be used for follow-ups to evaluate the increment in APV as the systolic function improves following treatment of CAD based on guideline management. It seems that the most useful indication of APV would be noninvasive cardiovascular risk stratification for better planning treatment of patients and detect high-risk ones.

Combined results of previous studies,^[9,12–15]^ showed a predictive value of APV in CAD with suitable specificity and sensitivity. It was also revealed that both AD and AS were significantly lower in CAD rather than non-CAD patients. Results of other similar studies confirmed this hypothesis in our meta-analysis. In Yildirim et al^[20]^ investigation, it was shown that APV level was lower in patients with severe risk factors in the past 10 years compared to those with moderate or low risk patients. It was revealed that atherosclerotic plaques in the thoracic aorta were indicators of CAD.^[21]^ Another report demonstrated that aortic plaques imaged by trans-esophageal echocardiography had a 93% sensitivity and 82% specificity for significant CAD.^[22]^ Atherosclerosis has been shown to escalate arterial resistance by a thickened and stiffened arterial wall.^[23]^ Bakirci et al^[23]^ demonstrated that a lower APV is one of the independent predictors of CAD severity in patients with stable CAD.

To the best of our evaluations, there are limited investigations in such field and there is scarce assessment of the SYNTAX score and arterial stiffness correlation. APV was significantly correlated with STNTAX score inversely, which demonstrates that as APV decreases, the SYNTAX score will be increase. SYNTAX score is an indicator of CAD severity^[24]^ and by using the APV index as an available marker, risk stratification of CAD patients would be easier, especially for transferring patients for invasive procedures.

Furthermore, the APV decreases due to the increased resistance that develops secondary to atherosclerosis in the descending thoracic aorta. Thus, the APV is an easy and simple echocardiographic parameter, which can be used to assess atherosclerosis in the thoracic aorta.

This was the first meta-analysis of the predictive effect of APV in CAD. Five articles were included in this study with a total of 490 patients. Our meta-analysis shows that APV has a predictive role in CAD using a simple and noninvasive procedure. However, each meta-analysis has its own limitations of methodology or research object. Because of lack of more studies in the field of APV in CAD, our included articles were limited.

## 5. Conclusion

A predictive value of APV in CAD was established with suitable specificity and sensitivity. Moreover, AD and AS indices were significantly lower in CAD and non-CAD patients. Nonetheless, higher quality and large-scale studies are expected to further quantify the predictive effect of APV. According to the results of this study, APV could be used as a good noninvasive tool for CAD screening.

## Author contributions

**Conceptualization:** Fereshteh Ghaderi, Hoorak Poorzand, Hedieh Alimi, Laila Bigdelu, Afsoon Fazlinezhad, Faeze Keihanian.

**Data curation:** Fereshteh Ghaderi, Shabnam Niroomand, Faeze Keihanian.

**Formal analysis:** Shabnam Niroomand.

**Investigation:** Faeze Keihanian.

**Methodology:** Shabnam Niroomand.

**Project administration:** Faeze Keihanian.

**Writing – original draft:** Shabnam Niroomand, Faeze Keihanian.

**Writing – review & editing:** Fereshteh Ghaderi, Faeze Keihanian.
